# Resveratrol Adjunct Therapy for Negative Symptoms in Patients With Stable Schizophrenia: A Double-Blind, Randomized Placebo-Controlled Trial

**DOI:** 10.1093/ijnp/pyaa006

**Published:** 2020-12-01

**Authors:** Areoo Samaei, Kamyar Moradi, Sayna Bagheri, Amir Ashraf-Ganjouei, Rosa Alikhani, Seiedeh Bentolhoda Mousavi, Farzin Rezaei, Shahin Akhondzadeh

**Affiliations:** 1 Psychiatric Research Center, Roozbeh Psychiatric Hospital, Tehran University of Medical Sciences, Tehran, Iran; 2 Psychosis Research Center, University of Social Welfare and Rehabilitation Sciences, Tehran, Iran; 3 Qods Hospital, Kurdistan University of Medical Sciences, Sanandaj, Iran

**Keywords:** clinical trial, primary negative symptoms, resveratrol, schizophrenia

## Abstract

**Background:**

Patients with schizophrenia can generally manifest a broad variety of primary negative symptoms. The current study aimed to assess the efficacy and tolerability of resveratrol add-on therapy in the treatment of negative symptoms in patients with stable schizophrenia.

**Methods:**

In a randomized, double-blind, and placebo-controlled setting, schizophrenia patients were assigned to receive either 200 mg/d resveratrol or matched placebo in addition to a stable dose of risperidone for 8 weeks. Patients were assessed using the positive and negative syndrome scale, the extrapyramidal symptom rating scale, and Hamilton Depression Rating Scale over the trial period. The primary outcome was considered as the change in positive and negative subscale score from baseline to week 8 between the treatment arms.

**Results:**

A total 52 patients completed the trial (26 in each arm). Baseline characteristics of both groups were statistically similar (*P*  > .05). Despite the statistically similar behavior of positive symptoms between the groups across time (Greenhouse-Geisser corrected: *F* = 1.76, df = 1.88, *P = .*180), the resveratrol group demonstrated greater improvement in negative, general psychopathology, and total scores (Greenhouse-Geisser corrected: *F* = 12.25, df = 2.04, *P < .*001; *F* = 5.42, df = 1.56, *P = .*011; F = 7.64, df = 1.48, *P = .*003). HDRS scores and its changes, ESRS score, and frequency of other complications were not significantly different between resveratrol and placebo groups.

**Conclusion:**

Adding resveratrol to risperidone can exhibit remarkable efficacy and safety in terms of management of schizophrenia-related negative symptoms.

**Significance Statement**

Schizophrenia is a prevalent, complex mental health disorder affecting neuropsychological domains. Schizophrenia patients can present negative symptoms such as loss of functional, verbal, and social abilities, which are usually complex in nature and resistant to conventional therapies. Hence, it is of great importance to use supplementary medications in addition to FDA-approved reagents to suppress negative symptoms as well. In this regard, we designed a randomized, double-blinded, placebo-controlled, parallel-group clinical trial and investigated the efficacy and safety of resveratrol add-on to risperidone. Notably, resveratrol has been recognized as a natural nonflavonoid polyphenol with antioxidative, antiapoptotic, antiinflammatory, and neuroprotective characteristics. As hypothesized, we identified the beneficial effects of resveratrol adjunct therapy on primary negative symptoms without any significant change in terms of safety in patients with stable schizophrenia. To the best of our knowledge, this study provides novel evidence regarding the beneficial roles of resveratrol supplementation in negative symptom treatment.

## Introduction

Schizophrenia is a complex, chronic mental health disorder causing a cascade of self-care, occupational, and cognitive impairments. The estimated prevalence of schizophrenia is 4.6–7.2 per 1000 individuals; meanwhile, it accounts for a remarkable portion of debility and costs among mental illnesses throughout the world (Saha et al., 2005; Rezaei et al., 2017; Hastrup et al., 2020). Schizophrenia can present a variety of neuropsychological disabilities, of which negative symptoms tend to be chronic and extremely debilitating, characterized by loss of various functions such as facial and emotional expressions, social communications, and verbal output (Akhondzadeh, 2001; Mitra et al., 2016). Despite the noticeable efficacy of the current antipsychotics in different perspectives of schizophrenia, the treatment of negative symptoms still remains challenging due to the complex nature and pathophysiology (Kirkpatrick et al., 2006; Chue and Lalonde, 2014; Millan et al., 2014).

A large body of evidence has reported the importance of cytokine disruption and propensity for the production of proinflammatory cytokines in schizophrenia that give rise to imbalance between neuroprotective and neurodegenerative factors (Potvin et al., 2008; Miller et al., 2011; Mansur et al., 2012; Upthegrove et al., 2014). In agreement, there is convincing evidence regarding the implication of dysregulated inflammatory pathways in negative symptoms (Kirkpatrick and Miller, 2013). Interestingly, oxidative stress that is intimately linked to a variety of pathophysiological processes, such as inflammation, has been proposed to contribute to the etiology of schizophrenia (Bitanihirwe and Woo, 2011). Another underlying mechanism predisposing schizophrenic patients to develop negative symptoms might be the alteration in action mechanisms of different neurotransmitters, including glutamate, dopamine, GABA, acetylcholine, and serotonin (Carlsson et al., 1999; da Silva Alves et al., 2008; Howes and Kapur, 2009; Brisch et al., 2014; Ghajar et al., 2018b).

On the other hand, nutritional supplements are of great interest in neuropsychiatric disorders (Ghajar et al., 2017, 2018b). Resveratrol (3,5,4’-trihydroxystilbene) is a natural nonflavonoid polyphenol that can be found in the skin of red grapes, red wine, and other natural dietary sources such as peanuts (Saiko et al., 2008). Resveratrol has wide beneficial effects, including antioxidative, antiapoptotic, antiinflammatory as well as neuroprotective effects (Kanthasamy et al., 2011). Resveratrol crosses the blood brain barrier due to its lipophilic characteristic (Rege et al., 2014) and can play a considerable role in preventing neuroinflammation and oxidation in the brain (Jeon et al., 2012b; Rege et al., 2013; Pineda-Ramirez et al., 2018). Moreover, resveratrol can generate neurotrophic factors by prompting astroglial cells, leading to the revival of disrupted dopaminergic neurons (Zhang et al., 2012). Altogether, it appears that resveratrol might positively affect schizophrenia patients and improve their symptoms, especially negative symptoms.

There is growing evidence regarding the use of resveratrol in many neurodegenerative diseases such as Alzheimer’s, Huntington’s, and Parkinson’s disease as it could exert neuroprotective effects against brain damage induced by toxins and inflammation (Jeon et al., 2012a; Rege et al., 2013; Giovinazzo and Grieco, 2015; Pasinetti et al., 2015). Beyond the common use, a number of previous double-blind, placebo-controlled, interventional studies in healthy adults have recommended a beneficial role for resveratrol in the treatment of cognition and memory impairment (Kennedy et al., 2010; Witte et al., 2014). Taken together, we assumed that resveratrol might be capable of improving negative symptoms. In this regard, we aimed to evaluate the efficacy and safety of resveratrol adjunct therapy in the management of negative schizophrenia-related symptoms in a double-blind, placebo-controlled setting for 8 weeks.

## Methods

### Study Design and Setting

An 8-week, randomized, double-blinded, placebo-controlled, parallel-group trial was conducted on patients with chronic stable schizophrenia at general psychiatric clinics, Roozbeh Psychiatric Hospital and Razi Psychiatric Hospital, from January 2018 to March 2019. After a complete description of the trial and its purpose, written informed consent was obtained from patients and their legally authorized representatives. They were notified of their right to withdraw from the trial at any time. The institutional review board of Tehran University of Medical Sciences approved the trial protocol (IR.TUMS.VCR.REC.1396.4183) that had been defined in full accordance with the latest version of Declaration of Helsinki (Declaration of Helsinki, as revised in Brazil 2013). The trial was registered in the Iranian registry of clinical trials (http://www.irct.ir; registration no.: IRCT20090117001556N103).

### Participants

Eligible participants (n = 60) were patients with 18–60 years of age who met the diagnostic criteria for chronic schizophrenia based on the fifth edition of Diagnostic and Statistical Manual of Mental Disorders and a minimum disease duration of 2 years (based on the Structured Clinical Interview for Diagnostic and Statistical Manual-5 Clinical Version (2013). In addition, participants must have had a minimum score of 15 in negative sub-score of positive and negative syndrome scale (PANSS) as well as clinical stability on a stable dose of risperidone for the last 8 weeks prior to the beginning of the study. Clinical stability was delineated as ≤20% change in the total score of PANSS on 2 consecutive ratings (every 2 weeks for a month) (Kay et al., 1987). Patients who have demonstrated any serious medical or neurological problem were excluded. Other exclusion criteria were intellectual disability based on clinical judgement of intelligence quotient < 70, alcohol or substance dependence during the last 6 months (except for nicotine and caffeine), suicide ideation, a score of 14 or above on the Hamilton Depression Rating Scale (HDRS), a score of ≥2 on the suicide item of HDRS, receiving electroconvulsive therapy during the last 3 months, lactation, pregnancy, history of allergy to risperidone or resveratrol, history of neurosurgery, or history of head trauma.

### Intervention

Eligible participants were randomly assigned to receive either risperidone (Risperdal, Janssen Pharmaceuticals, 4–6 mg) combined with resveratrol (ACER, Tehran, Iran; 200 mg/d) or risperidone (4–6 mg) combined with matched placebo capsule with an allocation ratio of 1. The dose selection for resveratrol was according to previous studies showing that high doses of the reagent may contribute to inhibition of cytochrome P450 isoenzymes and therefore, drug-drug interactions (Detampel et al., 2012). The medication regimen of participants was tightly controlled, since they were not allowed to use additional antidepressants, second antipsychotic agents, or mood stabilizers. Adherence to the medication plan was evaluated by comparing weekly the number of consumed capsules considering the report of medication intake from participants.

### Outcome

Schizophrenia-related psychopathological symptoms of participants were assessed using PANSS at baseline and at weeks 2, 4, 6, and 8 of the trial period. PANSS is a validated rating scale composed of 30 items that can be employed to measure positive (7 items), negative (7 items), and general (16 items) pathological symptoms of schizophrenia (Kay et al., 1987). In addition, the 17-item HDRS was used at baseline and week 8 to quantify depressive symptoms and their changes over the trial period (Hamilton, 1960). Both scales have been widely employed in previous clinical trials of schizophrenia in the Iranian population (Iranpour et al., 2016; Rezaei et al., 2017; Ghajar et al., 2018a).

The primary outcome of interest in the present study was defined as differences in PANSS negative subscale score reduction from baseline to week 8 between the treatment groups. Secondary outcome measures were considered as changes in other PANSS sub-scores between the treatment arms during 8 weeks of study.

### Safety

Extrapyramidal symptom rating scale (ESRS; part one: parkinsonism, dystonia, dyskinesia; sum of 11 items) was administered at baseline and at weeks 1, 2, 4, 6, and 8 to assess the extrapyramidal complications. ESRS can measure all drug-induced movement disorders irrespective of changes in psychopathology as measured by the PANSS (Chouinard and Margolese, 2005). The ESRS was previously applied in Iranian clinical trials demonstrating valid results (Iranpour et al., 2016; Rezaei et al., 2017; Ghajar et al., 2018a).

All eligible participants underwent thorough physical examination, 12-lid electrocardiography, complete blood counts, and liver function tests at baseline. Additionally, vital signs and body weight of each participant were monitored at each visit. Patients were educated to inform the research coordinates in case of any unexpected symptoms during the trial period. In addition to assessing extrapyramidal complications by ESRS, all cases were systematically asked whether they struggled with any kind of adverse effect during the trial, using open-ended questions followed by a 25-item checklist of side effects (Khajavi et al., 2012; Shahmansouri et al., 2014).

### Sample Size

The minimal sample size of 44 (22 in each group) was calculated based on assumption of a clinically significant difference of 3 on the PANSS negative subscale score, a SD of 3, a 2-tailed significance of 0.05, and a power of 90%. Considering a dropout rate of 20%, 54 participants were required. We enrolled 60 patients in the trial (30 in each group).

### Randomization, Allocation Concealment, and Blinding

Participants were randomly assigned to the treatment arms in a 1:1 ratio using a computer-generated code. The allocated group number was kept confidential in sealed opaque envelopes until data analysis. Resveratrol and matched placebo were identically encapsulated in terms of size, shape, color, and smell. Randomization, allocation, and blinding procedures were performed by an independent group not involved elsewhere in the study. Participants, outcome raters, medication distributor, referring physician, and research team were all blinded to the assignments.

### Statistical Analysis

Statistical analysis was carried out using the Statistical Package of Social Science Software (SPSS, version 24, IBM Company, Armonk, NY) for Windows. Graphs related to outcomes of interest were drawn using the GraphPad Prism 7 software (San Diego, CA). Compliance of variables with normal distribution was examined with the Shapiro-Wilk test and probability graphics. *P* values < .05 were considered statistically significant in data analyses. Categorical variables were reported in percentages (%) while continuous data were represented as mean ± SD. Baseline variables were compared between the groups using an independent *t* test with Levene’s test for equality of variance. The general linear model repeated-measures ANOVA analysis (corrected by Greenhouse–Geisser test in nonspherical cases) was used to compare scores of the PANSS, including of positive, negative, general, and total subsections, as well as ESRS score between the treatment arms over the course of the study. In addition, the quantity of the change in the PANSS scores from baseline to week 8 was measured using an independent *t* test with Levene’s test for equality of variance. Finally, the Fisher’s exact test was utilized to collate the frequency of side effects between the applied treatment strategies.

## Results

### Baseline Characteristics of the Patients

**Figure 1.**
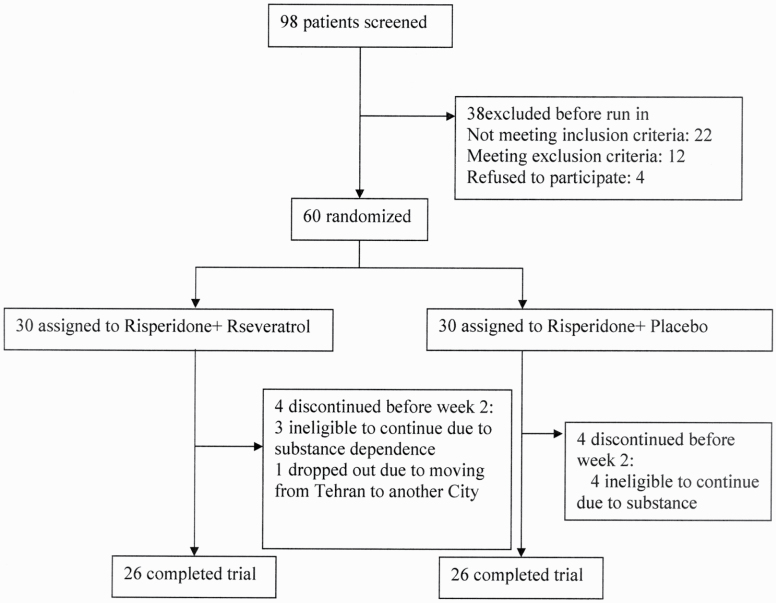
CONSORT flow diagram of the trial

Of 98 patients screened for eligibility, 60 patients were enrolled and randomly assigned to receive either risperidone plus resveratrol (n = 30) or risperidone plus placebo (n = 30) (Figure 1). Fifty-two participants completed the trial and underwent statistical analysis since 4 participants from each arm withdrew from the trial before the second week. Baseline positive, negative, general psychopathology, and total PANSS symptom scores, in addition to HDRS and ESRS values, were not significantly different between the 2 groups. The detailed description of baseline data is shown in Table 1. Participants showed favorable adherence to their treatment strategies.

**Table 1.** Baseline Characteristics of the Patients

Variable	Reseveratrol (n = 26)	Placebo (n = 26)
Gender, F(%)	10 (38%)	11 (42%)
Age, mean ± SD	34.73 ± 7.03	33.08 ± 5.48
Duration of illness, y, mean ± SD	11.46 ± 6.43	10.65 ± 5.20
Schizophrenia subtype		
Paranoid(%)	16 (61%)	17 (69%)
Residual(%)	4 (15%)	5 (19%)
Undifferentiated(%)	6 (23%)	5 (19%)
Marital status	Single: 16 (61%)	Single: 18 (69%)
	Married: 7 (27%),	Married: 5 (19%),
	Divorced: 3 (11%)	Divorced: 3 (11%)
Level of education	Less than high school diploma: 19 (73%), diploma: 4 (15%), illiterate: 3 (11%)	Less than high school diploma: 17 (69%), diploma: 7 (27%), illiterate 2 (7%)
Smoking	22 (84%)	23 (88%)
Baseline HDRS score, mean ± SD	9.84 ± 1.97	10.15 ± 1.97
Baseline ESRS, mean ± SD	0.77 ± 0.99	0.38 ± 0.70
Baseline PANSS		
Total score	70.08 ± 5.11	70.27 ± 4.21
Negative symptoms	17.69 ± 2.53	18.04 ± 1.75
Positive symptoms	20.58 ± 1.90	20.31 ± 2.17
General psychopathology	31.81 ± 2.40	31.92 ± 1.74

Abbreviations: ESRS, extrapyramidal symptom rating scale; F, female; HDRS, Hamilton Depression Rating Scale; PANSS, positive and negative syndrome scale.

### Negative Symptoms

**Figure 2.**
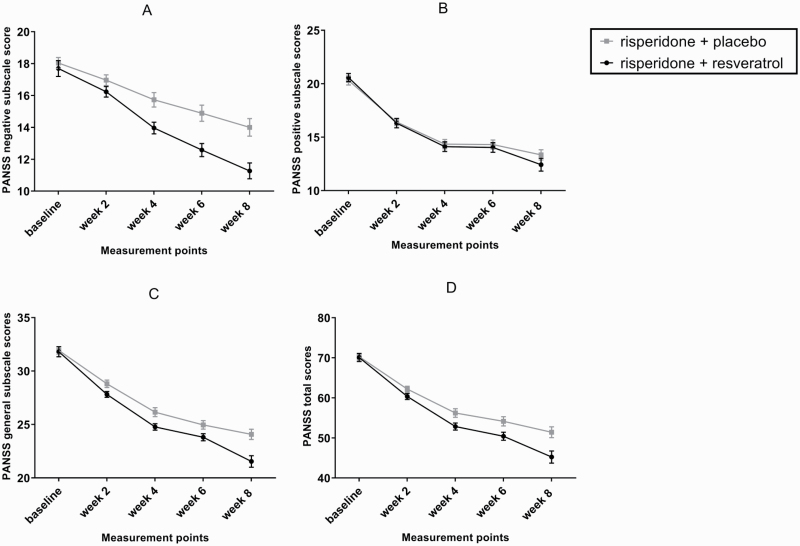
Repeated-measure ANOVA for comparison of the effects of 2 treatment strategies on the negative subscale of positive and negative syndrome scale (A), positive subscale of positive and negative syndrome scale (B), general psychopathology subscale of positive and negative syndrome scale (C), and positive and negative syndrome scale (D). Values represent mean ± SEM.

Repeated-measure analysis of ANOVA demonstrated significant effect of time (Greenhouse-Geisser corrected: *F* = 212.27, df = 2.04, *P < .*001) and time-treatment interaction (Greenhouse-Geisser corrected: *F* = 12.25, df = 2.04, *P < .*001), suggesting that the treatment groups had different behavior across time (Figure 2A). Table 2 gives a full image of reduction rates from baseline at measurement points for both arms. By week 8, the reduction rate from baseline score was significantly greater in the resveratrol group compared with the placebo group (MD [95% CI] = −2.38 [−3.67, −1.1], df = 50, t = −3.73, *P < .*001).

**Table 2.** Rate of Negative Symptoms Improvement From Baseline Over Time

Measurement points	Reseveratrol (n = 26)				Placebo (n = 26)			
	Paired differences							
	Mean	SD	t	*P*	Mean	SD	t	*P*
Week 2	1.46	1.58	4.71	.000	1.07	0.39	14.00	.000
Week 4	3.73	1.84	10.31	.000	2.31	0.84	14.05	.000
Week 6	5.11	2.06	12.63	.000	3.15	1.05	15.36	.000
Week 8	6.42	2.89	11.34	.000	4.04	1.51	13.64	.000

### Positive Symptoms

Repeated-measure analysis indicated significant effect of time (Greenhouse-Geisser corrected: *F* = 334.72, df = 1.88, *P < .*001). Nevertheless, the effect of time-treatment interaction was not significant (Greenhouse-Geisser corrected: *F* = 1.76, df = 1.88, *P = .*180), proposing similar changing trends of the groups in terms of positive symptoms (Figure 2B).

### General Psychopathology

Repeated-measure analysis exhibited significant effect of time (Greenhouse-Geisser corrected: *F* = 365.98, df = 1.56, *P < .*001). The effect of time-treatment interaction was also significant over the trial period (Greenhouse-Geisser corrected: *F* = 5.42, df = 1.56, *P = .*011), showing the differing influence of the treatment strategies on general psychopathology across time (Figure 2C). At week 8 of the study, the reduction rate from baseline score was significantly greater following resveratrol add-on to risperidone compared with the placebo adjunctive therapy (MD [95% CI] = −2.42 [−4.22, −0.62], df = 37.58, t = −2.72, *P = .*010).

### PANSS Total Score

The general linear model analysis of ANOVA was representative of differing efficacy of the administered treatment schedules over time, since it showed significant effect of time-treatment interaction (Greenhouse-Geisser corrected: *F* = 7.64, df = 1.48, *P = .*003) in addition to significance of time effect (Greenhouse-Geisser corrected: *F* = 448.81, df = 1.48, *P < .*001) (Figure 2D). At the end of the trial, the reduction rate from baseline score was significantly greater following resveratrol add-on to risperidone compared with the placebo adjunctive therapy (MD [95% CI] = −6.00 [−10.00, −1.99] df = 50, t = −3.01, *P = .*004).

### ESRS

**Figure 3.**
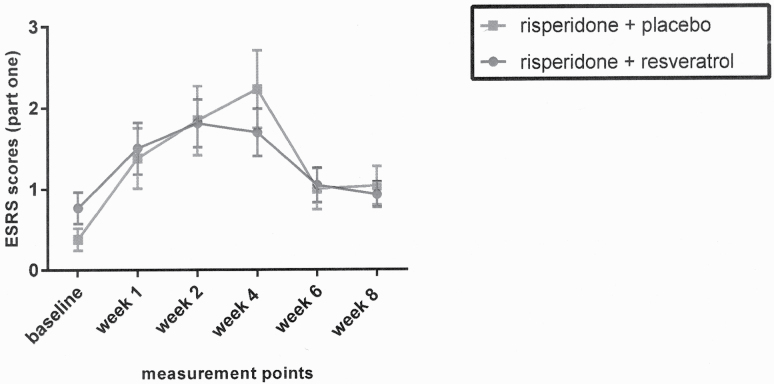
Repeated-measure ANOVA for comparison of effects of 2 treatments on the ESRS score. Values represent mean ±  SEM.

Although the effect of time was significant based on 2-way repeated-measures analysis of ANOVA (Greenhouse-Geisser corrected: *F* = 11.70, df = 3.11, *P < .*001), the groups showed no significantly different behavior across time, according to the analysis for the effect of time-treatment interaction (Greenhouse-Geisser corrected: *F* = 0.94, df = 3.11, *P = .*424) (Figure 3).

### Hamilton Depression Rating Scale

Similar to the baseline data, the treatment arms were not significantly different at the end of the trial (MD [95% CI] = −0.50 [−10.00, −1.99], df = 50, t = −1.07, *P = .*291), which is illustrative of statistically similar status of depressive symptoms between participants of the groups.

### Adverse Events

The details of detected side effects were listed in Table 3. All of the adverse events were tolerable with mild to moderate severity. The most frequent side effects were increased appetite and constipation in both of the groups. The frequency of complications other than extrapyramidal symptoms did not differ significantly between the groups (*P* > .05).

**Table 3.** Frequency of Adverse Events in the 2 Groups

Adverse events	Reseveratrol (n = 26)	Placebo (n = 26)	*P* value
Headache	4 (15%)	4 (15%)	1.000
Constipation	7 (26%)	8 (30%)	1.000
Diarrhea	5 (19%)	3 (11%)	.703
Fatigue	3 (11%)	4 (15%)	1.000
Nausea	3 (11%)	3 (11%)	1.000
Increased appetite	9 (34%)	7 (26%)	.764
Abdominal pain	6 (23%)	4 (15%)	.726
Nervousness	6 (23%)	7 (26%)	1.000

## Discussion

In this study, we demonstrated that adding resveratrol to the treatment regimen is well tolerated and improves negative symptoms of schizophrenia. Patients treated with resveratrol had significantly greater reduction of PANSS negative subscale from baseline, general psychopathology, and PANSS total score compared with the placebo group. Since negative symptoms can be affected by other manifestations such as positive and depressive symptoms, all patients were stabilized prior to the study to minimize the confounding factors. This claim is supported by the fact that baseline characteristics of both groups were not significantly different.

Management of negative symptoms is still a challenge for physicians because most antipsychotic agents do not have satisfactory effects on these symptoms (Erhart et al., 2006). Therefore, numerous studies have aimed to elucidate the pathophysiology behind negative symptoms, suggesting contribution to disruption in dopaminergic signaling pathways, GABAergic–glutamatergic balance, and inflammatory processes (Millan et al., 2014). In this regard, several clinical trials have been performed to investigate the beneficial role of adjunct therapies targeting these pathways. For instance, l-carnosine that is an antioxidant, significantly improved the negative score of schizophrenic patients (Ghajar et al., 2018b). In addition, Berk and colleagues demonstrated that adding N-acetyl cysteine to maintenance treatment of schizophrenia could significantly improve PANSS negative score (Berk et al., 2008). The combination of risperidone and celecoxib, which is a cyclooxygenase-2 (COX-2) inhibitor, also decreased the score of the negative as well as positive and general psychopathological symptoms (Akhondzadeh et al., 2007).

As mentioned earlier, resveratrol is phytoalexin compound that is found in various dietary sources, including grapes and peanuts, being known as an antiinflammatory, antioxidant, and neuro-protective agent. Since oxidative stress plays a prominent role in the pathogenesis of neurological diseases such as stroke and Alzheimer’s disease (AD), many researchers intended to evaluate its potential effects in these disorders. For instance, in patients with AD, treatment with resveratrol decreases the activity of matrix metalloproteinase-9, which is associated with the neurodegenerative process in AD patients (Turner et al., 2015). In this regard, resveratrol is proposed to decrease the level of matrix metalloproteinases in patients receiving delayed recombinant tissue plasminogen activator treatment, resulting in a better post-stroke outcome (Chen et al., 2016). Furthermore, caloric restriction has been shown to postpone the development of neurologic disorders by activating deacetylases such as sirtuins, which is also activated by resveratrol (Chung et al., 2010; Pasinetti et al., 2015). Though the cellular and molecular aspects of adding resveratrol to the treatment regimen of schizophrenic patients were not evaluated in our study, these mechanisms could explain some of the observed improvements resulting from the add-on.

Moreover, it has been assumed that hypoactivity of the dopaminergic system (particularly in the prefrontal cortex) is associated with negative symptoms in schizophrenia (Remington et al., 2011). In this regard, brain-derived neurotrophic factor has been associated with dopamine release in the brain (Narita et al., 2003), and its level is negatively correlated with negative symptoms in patients with schizophrenia (Tan et al., 2005). Interestingly, resveratrol administration has been shown to have antidepressant effects in an animal model of depression via the activation of brain-derived neurotrophic factor (Hurley et al., 2014). Furthermore, it has been shown that resveratrol has protective effects against glutamate toxicity by alternating the activity of Na^+^K^+^-ATPase and glutamine synthetase (Quincozes-Santos et al., 2014). This would prevent the dysregulation of N-methyl-d-aspartate receptors and intracellular Ca^2+^, which has been associated with the severity of negative symptoms in schizophrenic patients (Coyle et al., 2003; Coyle and Tsai, 2004).

Considering the immunomodulatory effects of resveratrol, previous studies have indicated that it prevents the production of interleukin (IL)-12 and tumor necrosis factor alpha by macrophages in addition to decreasing the production of IL-12 and interferon-gamma by lymphocytes (Gao et al., 2001). Available evidence suggests that patients with schizophrenia have higher levels of inflammatory cytokines such as IL-1, IL-6, and tumor necrosis factor-α (Potvin et al., 2008). Moreover, the severity of negative symptoms is positively correlated with increased levels of IL-6, IL-8, and C-reactive protein (Meyer et al., 2011). Since neurotransmitter (such as dopamine) interaction with inflammatory cytokines is indicated in the pathophysiology of schizophrenia, the above-mentioned pathways could also justify the significant reduction of negative symptom severity in schizophrenic patients receiving resveratrol as supplement therapy.

Although this is the first study, to our knowledge, evaluating the efficacy and safety of resveratrol as an add-on to risperidone in patients with chronic schizophrenia, a few studies had investigated the effects of resveratrol in animal models of schizophrenia and patients with schizophrenia. Interestingly, in a study by Zortea and colleagues, treatment with a 200-mg/d supplement of resveratrol in patients with schizophrenia did not have any significant effect on cognitive performance (Zortea et al., 2016). Although their study was of great value, it had some limitations such as a small sample size and short period of follow-up (Zortea et al., 2016). Moreover, Magaji et al. demonstrated that administering 200- and 400-mg/kg doses of resveratrol have significant antipsychotic and anxiolytic effects in the murine model of anxiety and schizophrenia (Magaji et al., 2017), which is in line with our results.

Although the current study has several advantages such as being novel, placebo controlled, double blinded, and adjusted with consideration to baseline variables, some limitations should be considered. Our follow-up period was relatively short and the sample size was small. Furthermore, the measurement of serum/cerebrospinal fluid biomarkers or neurotransmitters was not performed in order to propose a more precise justification for the observed results.

In conclusion, this study provides robust evidence considering the beneficial role of adding resveratrol to risperidone in the management of negative symptoms in patients with chronic schizophrenia. Further studies could elucidate the underlying mechanisms of supplement therapies such as resveratrol.
